# Coastal Waste Detection Based on Deep Convolutional Neural Networks

**DOI:** 10.3390/s21217269

**Published:** 2021-10-31

**Authors:** Chengjuan Ren, Hyunjun Jung, Sukhoon Lee, Dongwon Jeong

**Affiliations:** Software Convergence Engineering Department, Kunsan National University, Gunsan 54150, Korea; renchengjuan163@163.com (C.R.); junghj85@kunsan.ac.kr (H.J.); leha82@kunsan.ac.kr (S.L.)

**Keywords:** coastal waste, Faster R-CNN, deep convolutional neural network, environmental threat

## Abstract

Coastal waste not only has a seriously destructive effect on human life and marine ecosystems, but it also poses a long-term economic and environmental threat. To solve the issues of a poor manual coastal waste sorting environment, such as low sorting efficiency and heavy tasks, we develop a novel deep convolutional neural network by combining several strategies to realize intelligent waste recognition and classification based on the state-of-the-art Faster R-CNN framework. Firstly, to effectively detect small objects, we consider multiple-scale fusion to get rich semantic information from the shallower feature map. Secondly, RoI Align is introduced to solve positioning deviation caused by the regions of interest pooling. Moreover, it is necessary to correct key parameters and take on data augmentation to improve model performance. Besides, we create a new waste object dataset, named IST-Waste, which is made publicly to facilitate future research in this field. As a consequence, the experiment shows that the algorithm’s mAP reaches 83%. Detection performance is significantly better than Faster R-CNN and SSD. Thus, the developed scheme achieves higher accuracy and better performance against the state-of-the-art alternative.

## 1. Introduction

Improving people’s economy and quality of life have invariably increased human activities, which have brought about some negative effects, for example, environmental pollution [1]. Although environmental governance has never stopped, environmental pollution is still on the rise. According to the American “Science” magazine, by 2025, an estimated 250 million tons of waste will enter the ocean. Among them, coastal waste accounts for a very important proportion, and plastics are the main harmful pollutant [2]. The growth of coastal waste not only threatens marine life but also damages the living environment of surrounding residents [3]. Therefore, to degrade waste based on its nature and reduce environmental pollution, automatic waste classification and recognition are particularly important in the disposing of waste.

The vigorous development of computer hardware has laid the foundation for the amazing achievements of deep learning in the field of computer vision applications, including face detection [4,5], medical diagnosis [6], traffic safety monitoring [7,8], and smart agriculture [9,10]. For example, Qin et al. [11] developed the joint training model for face detection, which explains how the backpropagation is used in the training convolution neural network model cascade. To solve the occluding brought by the mask and sunglasses, Wang et al. [12] proposed a face detector FAN (Face Attention Network) that can effectively improve the precision of face detection in the occluded case. Shen et al. [13] introduced deep learning models to extract features, instead of traditional methods, by hand-designing features that can detect and classify, concerning the computer-assisted analysis of the image in medical images. The core of the algorithms can mine the different hierarchical feature representations from data, resulting in enhanced performance of medical analysis applications. Yao et al. [7] proposed the long-short-time memory model to predict freeway safety, and the naïve Bayes was employed to recognize image processing. Various stages of algorithm realization were studied, including data processing, model training, and implementation. Rahnemoonfar et al. [14] presented a simulated DCNN, which improves the Inception-ResNet model for fruit yield estimation. The experiment shows 93% in terms of test accuracy on synthetic and 91% on real images. From the works above, deep learning has been successfully applied to many fields and penetrated our lives.

Coastal waste destroys marine ecosystems and creates aesthetic discomfort. We can also frame the waste classification issue as an image classification task. Using deep learning to classify and identify waste is a fascinating research topic in computer vision, which also points out the direction of disposing of waste pollution on the coastal. ANH H. VO et al. [15] proposed a deep neural network, named the DNN-TC model, which was based on the ResNext model, to improve the trash classification performance. The experiments achieved the accuracy of 94% and 98% for NV-trash and Trashnet datasets respectively, which outperforms the performance of Densent121_Aral, RecycleNet, ResNext-101, and ResNet_Ruize on waste classification. Xu et al. [16] used the lightweight model and transfer learning to classify and identify waste by relocating and reconstructing MobileNetV2. The rebuilt network is employed for the extraction of classification features, and then, the SVM is considered as the model classifier to identify six categories of waste, which yields 98.4% in terms of accuracy for the TrashNet dataset. The paper also mentions that the improved model can conquer the problem of low data volume and over-fitting to realize high classification accuracy. Awe et al. [17] used Faster R-CNN to identify the different types of waste that were divided into paper, landfill, and recycling. The image dataset was produced by fusing 2–6 pieces of images of the TrashNet dataset on white background. The author fine-tuned the model by altering the last layers of the network and achieved 68% mAP. Fulton et al. [18] evaluated the performance of four state-of-the-art deep learning models, YOLOv2, Tiny-YOLO, Faster R-CNN, and SSD, on the marine debris dataset. A unique marine debris dataset was set up and used for the deep visual object detection task. However, the performance of those deep neural models is unsatisfactory in cases where the image contains small objects. To obtain more performance of the waste classification and detection, it is necessary to improve the model performance. The main contributions of our research are as follows.

First, we propose an improved deep convolutional neural network, based on Faster R-CNN [19], which is used to extract features and detect objects. Normally, the deeper the network layers, the lower the feature map resolution, the phenomenon results in the harder detection of small objects. To solve the issue and improve the accuracy of waste identification, we incorporate feature maps from the shallower, i.e., Conv4 layer, which has a higher resolution than the Conv5 layer. Their convergence makes the backbone network more invariant, equivariant, and more conducive to classification and identification. Second, the anchor mechanism is employed in the RPN network. In our model, instead of using default anchor parameters, we fit the anchor box scale, according to our dataset, to match objects and correct the contribution of objects in the loss function during the RPN training, which can improve model performance. Third, due to the lack of a sample, in the data pre-processing stage, we use the data augmentation technique to increase the diversity of original data and avoid model overfitting. Fourth, despite a currently large number of common image datasets, the waste dataset is rarely represented for object detection. To our knowledge, there is no publicly available coastal waste database. To continue future research work, we create the first public dataset in this field, named IST-Waste. Lastly, we verify the performance of the improved model on the dataset and show the meaningful enhancement performance over the state-of-the-art methods.

The rest of this study is organized as follows. Section 2 describes the related work of waste detection and classification. The background is covered in Section 3, i.e., the principle, advantages, and drawbacks of the Faster R-CNN. Then, Section 4 is dedicated to the improved model for the anchor box adjustment, data augmentation, and feature fusion. Section 5 presents the comparison of the experimental results and analysis. Finally, the conclusion is wrapped up by Section 6.

## 2. Related Work

Object classification and detection are some of the most basic tasks of computer vision. Nevertheless, research is relatively limited in the waste detection field. In our mind, the main reason for this phenomenon is primarily due to the scarce availability of public waste datasets. Therefore, we collect the IST-Waste dataset with 3000 images each annotated in the paper. To promote the next research in the area, we make the IST-Waste dataset publicly available. To the best of our knowledge, aside from the TACO dataset [20], including the 1500 dataset, IST-Waste is the unique public coastal waste dataset. Our work will be the first study in the classification and detection of coastal waste. Then, we briefly describe some classic works on waste classification, recognition, and segmentation, which are closely related to ours.

To resolve the issue of street litter pollution, Ping et al. [21] developed the deep neural network model to detect and classify the various type of street waste, such as leaves, tree branches, and so on. Additionally, the street waste images are collected and manufactured by the vehicle equipped with cameras and an edge station. Chen et al. [22] proposed an automatic grasping system for garbage classification, based on computer vision, where the RPN and the VGG model are used for classification and grabbing of the object. Ramalingam et al. [23] used the cascaded machine learning model, which combines CNN with SVM to detect and classify debris in floor-cleaning. The proposed method fields 95.5% accuracy and takes 71 milliseconds for the whole process of classification and recognition, which proves the approach is adaptive for arranging, in real-time, floor-cleaning applications. Jia et al. [24] presented an automatic inspection and cleaning table method using DCNN to detect the food litter on the table. High score confidence of classification is produced for each type of waste, such as liquid and solid. The built scheme is compared with Faster R-CNN Resnet and SSD models in the paper, which verifies the validity for the HSR robot. Toğaçar et al. [25] introduces the comprehensive method, based on the AutoEncoder network and feature extraction of the CNN model, with the SVM as a classifier to classify waste. The RR algorithm is used to reduce the number of features and disclosed valid features. The results show that the ResNet-50 model expresses the best waste classification performance comparison with AlexNet and GoogLeNet on two datasets.

The works involve the segmentation component. Bai et al. [26] presented the robot for automatically cleaning garbage, with two-stages CNN, on the grass in places such as playgrounds or parks. Firstly, the authors implemented waste segmentation based on the SegNet model on the ground without human involvement. Then, the famous ResNet model was employed for waste classification. To solve the problem of waste location from RGB and depth image, Wang et al. [27] developed the novel waste segmentation structure that fuses depth and intensity reasoning, which does not desire target-level annotations. The improved CRF model extracts the last segmentation results with depth-level, appearance-level, and pixel-level information. They collected the MJU-Waste dataset, which is the first public dataset for waste segmentation.

Although each of these papers surveyed above have made certain achievements in waste classification, detection, or segmentation. The natural properties of waste itself bring many difficulties to the research work in the area, such as the variety of waste, millions of shapes, complex and irregular stacking shapes, and even the phenomenon of waste decay and mutilation. Therefore, researchers sometimes have to look for a balance between model performance and speed.

## 3. Background

In the era of deep learning, image classification and object detection began to evolve at an amazing speed. In this section, we look back to well-known deep convolutional neural networks, namely Faster R-CNN. 

### 3.1. Faster R-CNN

R-CNN [28] permits the extraction of many object candidate detection boxes by selective search. Then, each candidate area is cropped to the fixed-size image before being fed into the network to extract features. Last, the SVM is the classifier to detect the objects. Although R-CNN has achieved good performance in object recognition, its shortcomings cannot be ignored: due to the inability to share feature calculations, the network generates a large number of computations redundancy, which results in a very slow detection speed. In 2015, Fast R-CNN [29] was proposed to conquer the fatal defect of the R-CNN, which comes true for the shared features between proposals, and integrated the detector and bounding box regressor into model configurations. However, the speed of network detection is still slow with the proposal detection strategy. 

**Figure 1:**
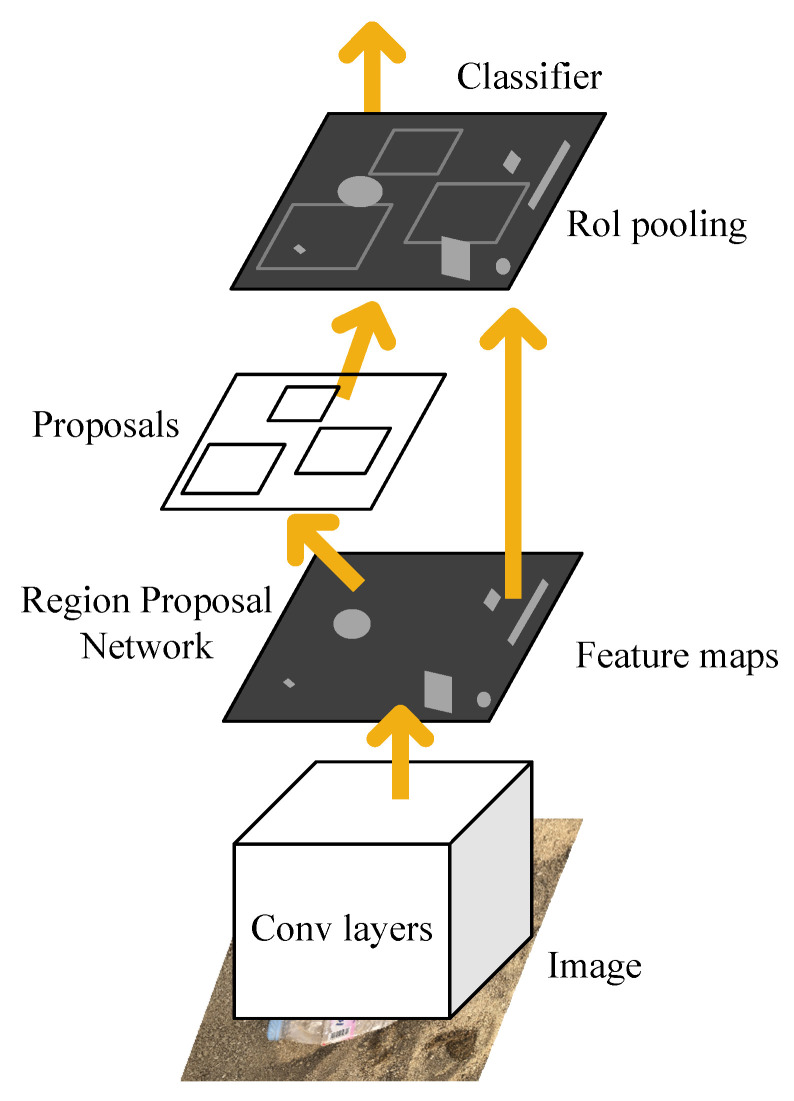
The structure of the Faster R-CNN.

The same year, Ren et al. presented Faster R-CNN [19] that realizes nearly real-time object detectors and is the first end-to-end model in deep learning. The structure of the Faster R-CNN is shown in Figure 1. The proposed Region Proposal Network (RPN) and the last feature map of the Fast R-CNN are closely related to predict object position and confidence scores. Faster R-CNN firstly uses a set of basic Conv + Relu + pooling layers to extract image feature maps. The feature maps are shared for subsequent RPN and full connection layer. RPN network is used to generate region proposals. This network determines which anchors are positive or negative by softmax and then bounces box regression correct anchors for precise proposals. Furthermore, the region of interest (RoI) pooling is employed to accelerate the speed of model detection. This layer collects the input feature maps and proposals, integrates this information, extracts the proposal feature maps, and sends them to the subsequent full connection layer to determine the target category. Last, the SVM technique is discarded, and the final classification is implemented using fully connection. Because of the shared feature in the model, the model can greatly reduce the training and testing time. 

### 3.2. Limitation for Small Object Detection 

Small object detection is a challenge and fundamental computer vision task that recognizes instances of small physical size objects in images. There are several reasons why small objects are difficult to detect in images [30]. Firstly, because of the small physical size of the object, it is difficult to distinguish it from the background or similar classes. Secondly, the smaller objects are more likely to be obscured and have more possibilities for the location, which results in difficult detection. Besides, object detection has been mainly focused on the study of generic objects, so research work committed to small object detection is few. As a result, research on small objects has developed slowly. Multi-scale feature learning has been widely applied to advance the performance of small object detection, including, for example, single feature maps, integrated features, feature fusion, and feature pyramid networks. Another way to solve the problem is data augmentation during the training phases of the network, which can be dependent on increasing the number and type of small objects samples in the dataset.

## 4. Our Approach

**Figure 2:**
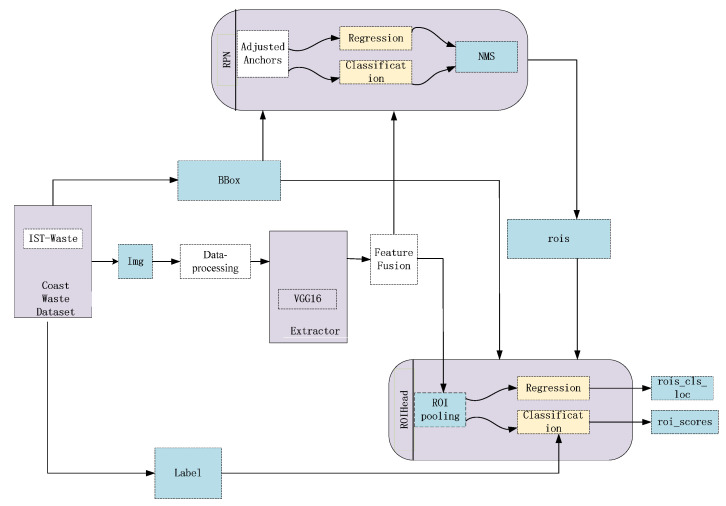
Structure of our approach.

This part mainly describes the proposed method for coastal waste recognition. Feature fusion, RoI Align, correction of the anchor boxes, and data augmentation are employed to achieve richer semantic presentation. The ultimate goal of our approach is to obtain effective and accurate context information to improve the detection performance of the Faster R-CNN. The detailed structure of our approach is shown in Figure 2. In general, the proposed method still consists of the RPN and Fast R-CNN. We import more details into the Faster R-CNN to train the proposals. In the data pre-processing stage, we use data augmentation to increase the number of samples and avoid model overfitting. Secondly, after inputting the coastal waste image, the VGG16 model is used to extract image features. Then, the fusion feature map, with higher resolution and high-level semantic information from the fourth and the fifth feature maps, is more conducive to the detection of small objects. At the same time, it also serves as an input for subsequent models. Next, we optimize anchor boxes by the clustering algorithm, which is fitted for better coverage of the size of objects according to the distribution of the coastal waste. RoI Align can reduce proposal deviation.

Similar to the original Faster R-CNN, the multi-task loss function also contains location loss and classification loss in our model. In the RPN network, the produced anchor is only divided into foreground and background, labeled 1 and 0, respectively. The classical binary cross-entropy is used to calculate the classification loss. In Fast R-CNN, multiple-classification cross-entropy is employed to compute the loss. In the regression section, the loss is counted only in the foreground. The model total loss is defined as:

(1) $$L\left(p_{i,}t_{i}\right)=\frac{1}{N_{cls}}\sum_{i}L_{cls}\left(p_{i,}p_{i}^{*}\right)+\lambda \frac{1}{N_{reg}}\sum_{i}p_{i}^{*}L_{reg}\left(t_{i,}t_{i}^{*}\right)$$

The objective of the training is to iteratively minimize the average empirical, where $L_{cls}\left(p_{i,}p_{i}^{*}\right)$ is prediction of the classification loss, $p_{i}^{*}L_{reg}\left(t_{i,}t_{i}^{*}\right)$ is prediction of the regression loss, *λ* is the balance parameter.

We use Stochastic Gradient Descent which is a classic optimization algorithm adopted in convolutional deep networks to update model weights.

(2) $$w_{i+1}=\mu w_{i}-\alpha \nabla J\left(w_{i}\right)$$

where *α* is the learning rate, *μ* is the momentum weight for weight $w_{i}$, and ∇ is the partial derivative operator.

### 4.1. Region Proposal Network (RPN)

**Figure 3:**
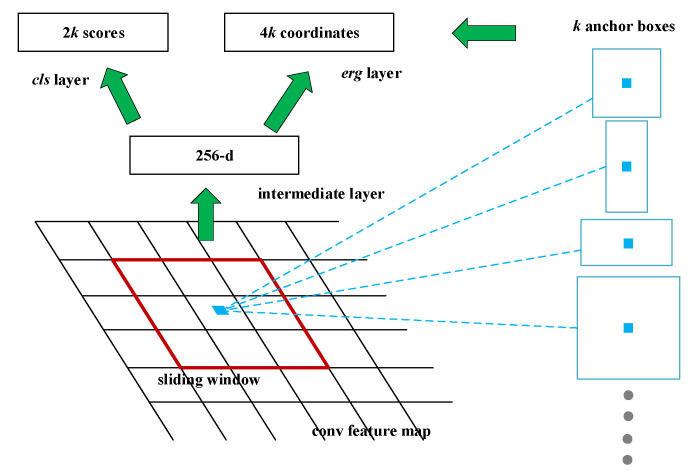
Structure of the Region Proposal Network (RPN).

The main innovation of Faster R-CNN belongs to RPN and is responsible for predicting object bounding box with anchor mechanism and score for the object. The essence of each score is to determine whether there are objects in the proposal regions. Figure 3 represents the framework of the RPN. The shared feature map of Faster R-CNN is mainly used for RPN and participates in the operation of RoI pooling. A convolution operation of 3 × 3 is performed on the feature map, and finally, the intermediate layer, with 256 channels, is obtained. Every center of the feature map corresponds to an area of the original image and is covered by the k anchor boxes. The whole anchor consists of anchor boxes with different scales and aspect ratios, describing objects of various sizes. 

**Figure 4:**
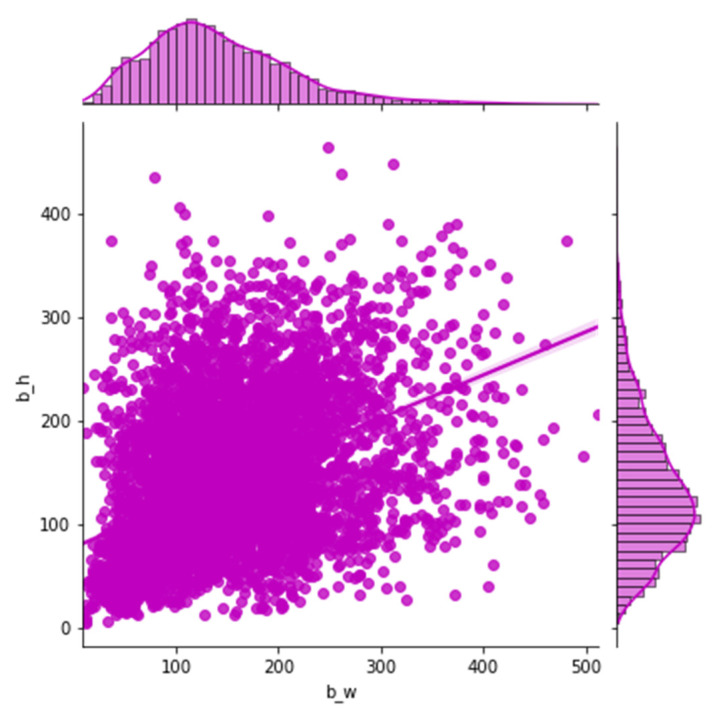
The width and height distribution of the box from our dataset.

**Figure 5:**
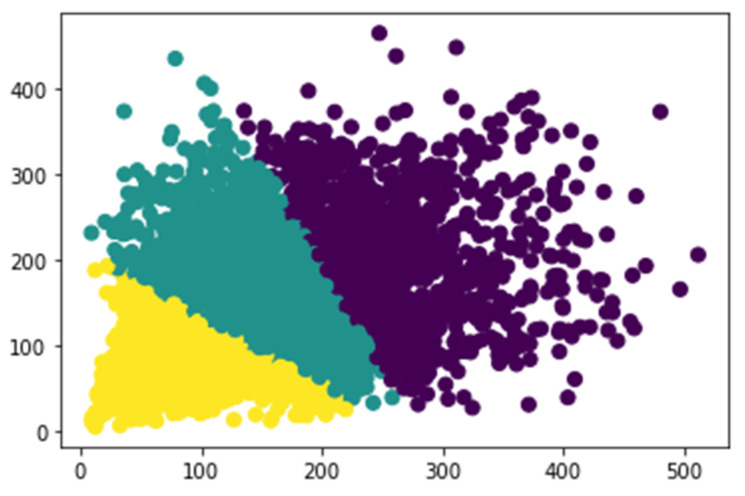
K-means clustering results from our dataset.

Most of the previous networks have used specific heuristics to decide anchor values. For example, there are nine anchor boxes in the standard Faster R-CNN, which are based on the size of hand-picked values, including three scales (128 × 128, 256 × 256, 512 × 512) and three aspect ratios (1:1, 1:2, 2:1). In practical applications, objects come in all shapes and sizes. If we still quote the default size of the anchor box, which has a passive impact on the performance of the training model, objects of various sizes for different datasets, settling adaptive size, and the number of anchors can accelerate the model convergence speed and advance the detection accuracy. In our works, instead of using default values in the original Faster R-CNN, we apply k-means clustering on our dataset, inspired by YOLO [31], to automatically adopt anchor box size. To balance the computational complexity and the accuracy of the model, three basic size boxes are selected for clustering in the initialization. It is the width and height distribution of the box from our dataset in Figure 4. The k-means clustering result according to our samples is shown in Figure 5. Firstly, the three initialized samples are selected as the initial cluster center, including the distance between each sample in the dataset. Next, the three cluster centers are calculated and divided into the corresponding class of the cluster center with the smallest distance. Finally, its cluster center is recalculated for each category until the minimum error result is obtained.

Eventually, we assign three aspect ratios {1:2, 2:1, 1:3} and scales {128 × 145, 196 × 212, 256 × 378} of the anchor boxes, which can take into account the coastal waste of different scales in our dataset. Thus, nine anchor boxes are generated at every center of the feature map. Then, 36 box regression and 18 box classifications are produced in the RPN for every region proposal.

### 4.2. RoI Align

In common two-stage detection frameworks (such as Fast-RCNN, Faster-RCNN, and RFCN), RoI pooling is used to pool the corresponding area in the feature map into a fixed-size feature map, according to the position coordinates of the proposal boxes, and conduct subsequent classification and box regression operations. Since the position of the proposal box is usually obtained by model regression, it is a floating-point number, and the pooled feature graph requires a fixed size. After the above two quantifications, the proposal boxes, at this time, have a certain deviation from the original regression position, which affects the accuracy of detection or segmentation, especially for small object detection. To advance the issue, the RoI Align algorithm is adopted to get the feature map of the rich information. The quantization operation is canceled, and the bilinear interpolation method is used to obtain the image values of the pixels, whose coordinates are floating-point numbers, so the whole process of feature aggregation can be transformed into a continuous operation. There are three steps for RoI Align:

(RoI division): The candidate regions are divided into k × k cells and each cell is not quantified.

(Interpolation): Interpolating the values of all sampling points (each grid s × s points).

(Max pooling): Finding the maximum value of all s × s sampling points in a grid.

### 4.3. Data Augmentation

Training dataset has a significant impact on detection model performance. That’s because the only source for detection model learning features is from training data. The lack of training data is the first key problem that researchers should tackle. Especially for the small objects, the research found that the computed IoU between the predicted anchor boxes and the ground-truth boxes is much lower than expected. Data augmentation strategy can deal with the issue and bring stronger generalization ability to the model. In coastal waste detection, we use data augmentation, including cropping, rotating, and scaling to produce auxiliary samples of waste. It can not only effectively alleviate the overfitting of the model, but it can bring richer the feature of the model. The accuracy of small object detection can also be advanced by expanding the categories and numbers of small object samples during training.

### 4.4. Feature Fusion Layer

**Figure 6:**
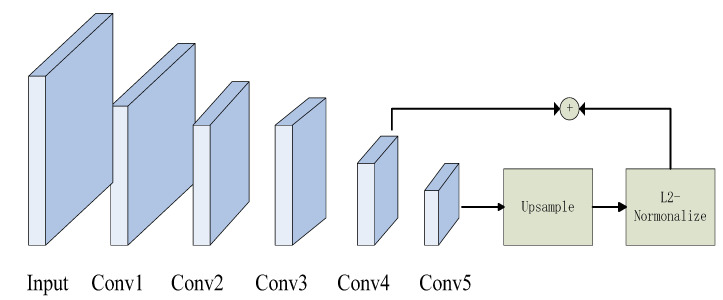
The structure of multi-scale feature map fusion.

Instance object detection has always been a difficult task in general object detection. Cigarette butts, glass residue, and bottle caps in the obtained samples are sometimes low-resolution. The VGG16 is regarded as the backbone in the Faster R-CNN, which owns five feature maps. The whole model only uses the fifth feature map to join the subsequent work. Therefore, it is difficult for the state-of-the-art Faster R-CNN to recognize small objects. The first reason is that the single-layer feature map represents incomplete image information. Another reason is that the Conv5_3 has a large receptive field. It can capture a wide range of contextual information and ignore the smaller ones. We then fuse the convolutional feature maps, Conv4_3 and Conv5_3, to enhance semantic features. The structure of the multi-scale feature map is shown in Figure 6. The size of every feature map is different in the model. We adjust the size of the Conv5_3 to match the Conv4_3 by up sampling the Conv5_3. Then, the L2-normalization output of the two layers [32] is concatenated to utilize as the input for the RPN.

## 5. Experiments

Apart from describing the experimental setup, such as Nvidia Tesla T4, Pytorch 1.0, and Python 3.6, this section analyzes the experimental performance of the networks. The proposed network should be able to detect and classify different types of coastal waste. In the training deep learning model, the learning rate is a very important parameter, which determines whether the objective function can be linked to the local minimum. To achieve local optimum, the learning rate gradually decreases with the reduction in loss (detail information in Section 5.2). In our model, the Stochastic Gradient Descent is used. The initial learning rate is $10^{-3}$. 

### 5.1. Dataset Construction

**Figure 7:**
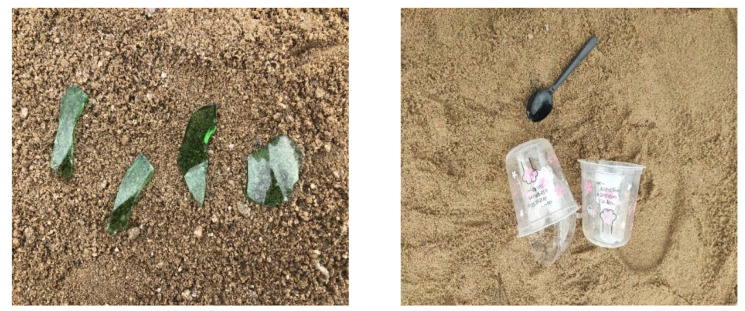

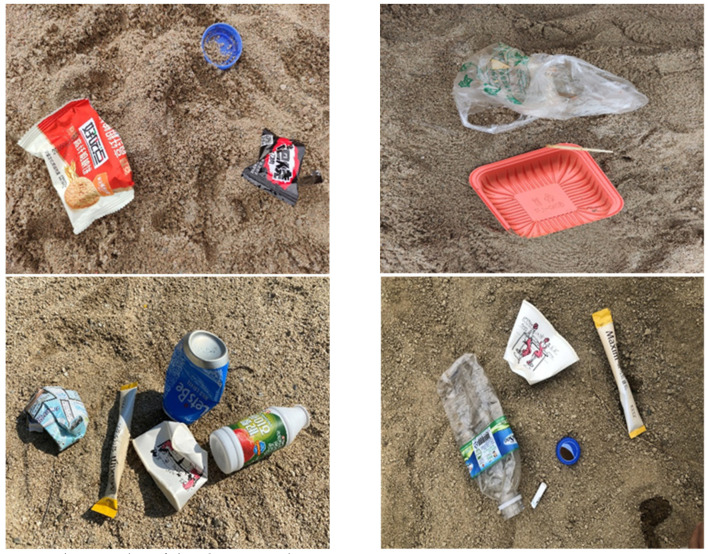
The samples of the IST-Waste dataset.

Since there are no public datasets in coastal waste recognition studies, the data used in the paper were obtained by camera shooting. There are 3000 images in the IST-Waste dataset, including six classes (plastic, glass, paper, butt, metal, and wood). The image format is jpg format. Depending on the actual scenario under the diverse weather (rain or sunny), light (brightness or shadow), and blocked by other, the number of classes varies, and even a single image can contain multiple categories with different shape and size. Plastic and paper appear more frequently in daily life than other classes, so they have more targets than any other class. The number of objects in the dataset for every class is presented in Table 1. The original image varies in size, from 1024 × 1024 to 3000 × 4000. Some samples of the IST-Waste dataset are shown in Figure 7. During the training phase, we divided the samples into training and testing parts, accounting for 80% and 20%, respectively. To effectively alleviate model overfitting and bring stronger generalization ability to the model, we use the data augmentation technique to increase samples.

### 5.2. The Experiment Results and Analysis

The evaluation metric of the object detection commonly contains the mean Average Precision (mAP), the Average Precision (AP), F1 score, Recall rate, and so on. The mAP is considered the average of AP of all object categories. Thus, we use mAP and F1 scores as the authoritative metric to evaluate the performance of our model. Table 2 introduces typical and related evaluation metrics.

Measuring the probability that the positive class detected by the model is indeed a positive class.

(3) $$\mathrm{Precison}=\frac{TP}{TP+FP}$$

(4) $$\;\mathrm{Recall}=\frac{TP}{TP+FN}$$

(5) $$F1=\frac{2\times Precision\times Recall}{Precision+Recall}$$

The main direction of our experiment analysis is as follows. On the one hand, we try to evaluate the performance of the improved Faster R-CNN model in coastal waste object detection. On the other hand, we are going to look at how the different tricks affect the model performance, especially for small object detection. To achieve the goal, we did a couple of different experiments. We set the learning rate to $10^{-3}$ for the beginning 70k iterations, then keep training $10^{-4}$ for 20k iterations and $10^{-5}$ for the final iterations. Batch size smaller than 12, and trained on 4GPUs, can get stable results in batch normalization and accuracy. This experiment still follows the input rules of Faster R-CNN, limiting the minimum edge to 600 and the maximum edge to 1000. The size of the final input image is 480 × 800. It took us about one day to train the model from iterative experiments to determine the input image size to obtain the final parameters.

**Figure 8:**
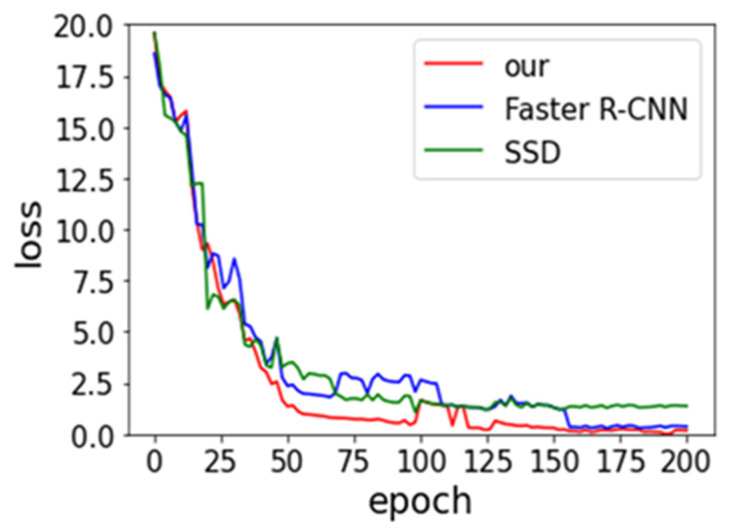
Effects of the number of epoch on the training loss.

**Figure 9:**
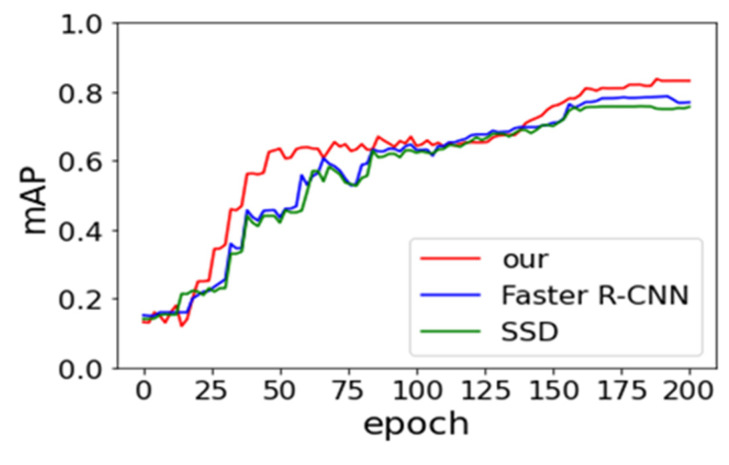
Effects of the number of epoch on the overall accuracy of the test set.

Faster R-CNN and SSD models are derived from Ren et al. [19] and Liu et al. [33] research in our work. We compare their performance of three models in order to be fair. The same backbone network, with the data augmentation approach, is utilized. The batch size and SGD remain consistent. Figure 8 and Figure 9 provide the experimental result of loss vs epoch and accuracy vs epoch on IST-Waste. From the below two figures, we can observe that our method achieves the lowest loss and the highest accuracy, but the rate of convergence is lower than others. The SSD model shows the fastest speed. One of the possible reasons is the SSD belonging to the end-to-end training model, which requires small computing resources. 

The test results are shown in the later Appendix A. From the test results, we can see that each waste can be detected very well, and even small objects show promising results. However, there is a large number of objects stacked in the image, accompanied by occlusion or distortion, the detection effects are affected. Especially when the object is deformed or decayed, it is also difficult for the human eye to judge. This is still a challenge for object detection.

Table 3 provides the assessment result of the proposed algorithm compared to SSD and Faster R-CNN in terms of accuracy per class. The result shows that our model gets better performance than others. For the category of the small objects, such as butt and wood in IST-Waste, the accuracy of our method is significantly higher than others, the recognition rate of the wood category from 55.2% to 63.3%, the butt recognition rate from 52.9% to 71.5%. One of the reasons is that the feature fusion strategy is adopted to acquire richer context information and increase local and global semantic information for object detection. Another reason is that, although small objects have a small size, RoI Align is used to cancel two quantization errors, which is beneficial to small object detection. The adaptive anchor boxes, by clustering, used on our model can better fit the size of the objects, which boosts the accuracy of the classes. Although feature fusion and other technologies are added, the number of parameters is not increased. The number of parameters in our model is similar to that in Faster R-CNN.

We report the overall ablation experiments in Table 4. On the Faster R-CNN baseline, the augmentation, adjusted anchor boxes, RoI Align, and feature fusion are gradually increased. The ablation study on our dataset is implemented with the same parameter setting for a fair comparison. The “Faster R-CNN” notes the standard Faster R-CNN model. Data augmentation is used to avoid model overfitting, such as cropping, rotating, and scaling. When we substituted RoI Align for RoI pooling, 81.4% mean average precision is obtained, an increase of 2.2 mAP and 1.0 F1 scores compared to the standard Faster R-CNN. Because of the lack of a sample, every ablation sub-experiment contains data augmentation technology. When adding layer fusion and adjusted anchor boxes, we achieve 81.8% and 81.6 mAP, respectively, which proves the effectiveness of the increased tricks. Finally, we directed plentiful experiments to verify the effectiveness of our proposed method. 

## 6. Conclusions

Object instance detection is always a difficult problem in general object detection. Coastal waste often contains a lot of small objects, such as cigarette butts, scraps of paper, broken glass, bottle caps, etc. In the paper, we proposed a deep neural network, based on Faster R-CNN, to detect coastal waste. We aimed at synchronizing several options to improve the standard Faster R-CNN performance. Detecting small objects could be addressed by fusing high-resolution features with high-dimensional features from the low-resolution image. Moreover, generating anchor boxes, according to the size of our dataset, was conducive to improving the performance of the model. Besides, RoI Align instead of RoI pooling to solve position offset could also effectively boost the performance of automated coastal waste detection. Data augmentation brought into the model avoided the overfitting phenomenon. Eventually, the experimental results showed that the developed deep learning model has obtained a relatively good accuracy, which has met the requirements of coastal waste detection and revealed the great potential in the related topics. 

We still face some challenges, such as object deformation or decay of the object, data annotation, and model selection. In future work, we will collect more samples to exploit the model to produce more detection performance. At the same time, it will be worth researching direction for the improvement of model speed.

## Figures and Tables

**Table 1:** The number of objects in the dataset.

No	Classes	The Number of Images
1	Plastic	4757
2	Metal	369
3	Paper	1740
4	Butt	389
5	Wood	367
6	Glass	248

**Table 2:** Related metrics for object detection.

	Positive (Predictive)	Negative (Predictive)
Positive (Truth)	True Positive (TP)	False Negative (FN)
Negative (Truth)	False Positive (FP)	True Negative (TN)

**Table 3:** The results of algorithms (%).

	Plastic	Metal	Paper	Butt	Wood	Glass	mAP
Faster R-CNN	87.5	87.4	87.1	65.4	58.6	88.8	79.2
SSD	86.3	84.7	87.2	52.9	55.2	93.7	76.6
Our	89.1	89.7	93.2	71.5	62.3	92.3	83.0

**Table 4:** Ablation experiments (%) (√ notes to introduce this technology in the model).

	Faster R-CNN					
Data augmentation		√	√	√	√	√
Adjustive anchor boxes					√	√
Layer fusion				√		√
RoI Align			√			√
Test mAP	79.2	80.2	81.4	81.8	81.6	83.0 (our)
F1 Scores	76.9	77.5	77.9	78.2	80.4	82.3 (our)

**Figure A1:**
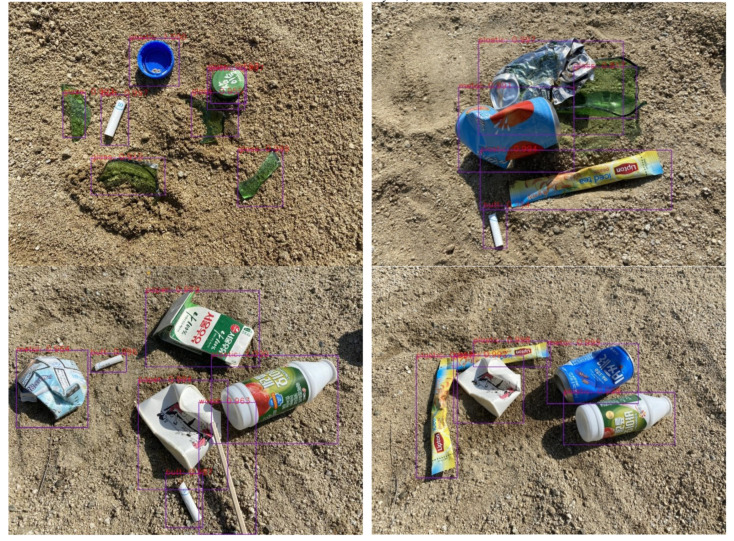

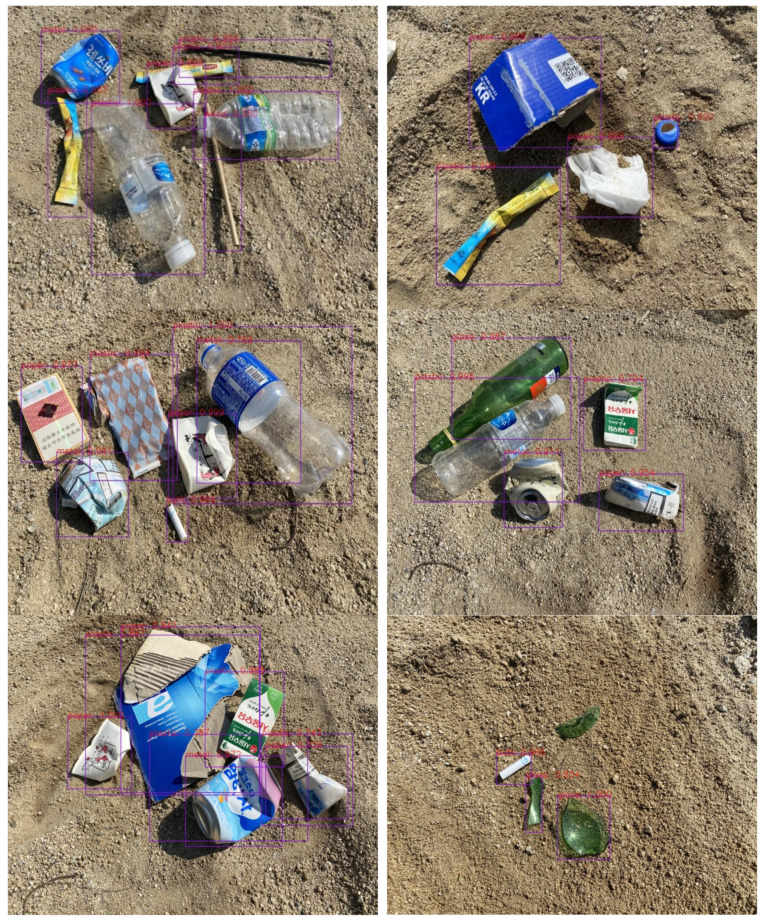

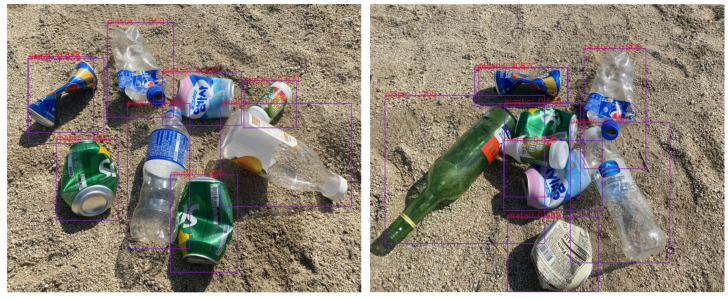
The test results on our model from the IST-Waste dataset.

## Data Availability

Not applicable.

## Abbreviations

The following abbreviations are used in this manuscript:

SSD	Single Shot MultiBox Detector
YOLO	You Only Look Once
SVM	Support Vector Machines
RPN	Region Proposal Network
TP	Truth Positive
FP	False Positive
FN	False Negative
CNN	Convolutional Neural Network
RoI	Region of Interest

## Appendix A

(See Section 5.2 for detailed analysis)
